# Effectiveness of intravitreal chemotherapy-assisted endoresection in monocular patients with group D retinoblastoma

**DOI:** 10.1186/s12885-020-07314-1

**Published:** 2020-08-26

**Authors:** Xiling Yu, Xueke Li, Yue Xing, Siduo Lu, Silvia Tanumiharjo, Jin Ma

**Affiliations:** Zhongshan Ophthalmic Center, State Key Laboratory of Ophthalmology, Sun Yat-sen University, Guangzhou, China

**Keywords:** Refractory retinoblastoma, Intravitreal chemotherapy, Endoresection, Pars plana vitrectomy

## Abstract

**Background:**

This study aimed to determine the efficacy and complications of intravitreal chemotherapy-assisted endoresection for refractory International Classification of Retinoblastoma (ICRB) group D retinoblastoma in monocular patients.

**Methods:**

In this retrospective case series, intravitreal chemotherapy-assisted endoresection by pars plana vitrectomy was performed in 11 eyes with refractory ICRB group D retinoblastoma unresponsive to standard therapies in monocular patients.

**Results:**

Across a mean follow-up period of 42.7 months, globe salvage was attained in all 11 eyes (100%). There were no cases of extra-ocular tumour seeding or remote metastasis. In 9 eyes (81.8%), tumour control was achieved with one pars plana vitrectomy; in 2 cases (18.2%), repeated treatment, such as laser therapy, intravitreal chemotherapy or a second pars plana vitrectomy, was needed. Retinal reattachment was achieved in all 4 eyes (100%) with previous retinal detachment. Four eyes (36.4%) required subsequent cataract surgery due to secondary cataract. Ten eyes (90.9%) had improvement in best-corrected visual acuity at the last follow-up.

**Conclusion:**

Intravitreal chemotherapy-assisted endoresection appears to be a safe and effective globe-salvaging method for refractory group D retinoblastoma. It is a promising alternative to enucleation and a supplementary therapeutic strategy for those unresponsive to standard therapies, especially for the monocular retinoblastoma patients.

## Background

Over the past two decades, the management of retinoblastoma has dramatically changed from eye-sacrificing methods to eye-preserving alternatives [1]. Eye preservation can be achieved in as much as 90–100% of eyes classified as group A, B or C retinoblastoma, but it is still challenging in group D and E retinoblastoma classified according to the International Classification of Retinoblastoma (ICRB) criteria [2]. Recent research has suggested that it is safe to attempt eye-preserving methods for group D cases, while enucleation is still recommended for group E cases [3]. Recent studies have shown some progress in the globe salvage of group D retinoblastoma with the single or combined use of intravenous chemotherapy (IVC), intra-arterial chemotherapy (IAC) and intravitreal chemotherapy (IViC), but persistent or recurrent vitreous and subretinal seeding can still occur in some refractory cases [4]. Eye preservation is especially important for monocular patients for whom the other affected eye has been previously enucleated for unmanageable progressive retinoblastoma and who require the remaining eye to retain visual function.

IViC was first introduced in 2003 [5], and it has become one of the most effective treatments against the vitreous seeding of retinoblastoma [6]. Several studies have proven that IViC has increased the eye-preserving rate in group D retinoblastoma [3, 4, 7]. However, the recurrent refractory vitreous and subretinal seeding of the tumour remains a major challenge of this method [6]. The efficiency of IViC is reduced when there is a high burden of vitreous seeding, as tumour growth cannot be controlled even with repeated IViC. Furthermore, sight-threatening tumour-related complications, such as persistent retinal detachment or vitreous haemorrhage, cannot be solved through IViC.

Theoretically, endoresection by pars plana vitrectomy (PPV) is the ideal way to eradicate a tumour and solve vitreoretinal complications, but its use in the treatment of retinoblastoma has been controversial due to the high risk of metastasis, orbital seeding and extraocular extension [8]. However, the recent success of IViC has raised the possibility of surgical intervention again [9, 10]. In this study, we attempted IViC-assisted endoresection in refractory monocular ICRB group D retinoblastoma patients. Intraocular surgery was carefully executed and combined with effective local chemotherapy and a series of safety measures. We evaluated the therapeutic effect and complications with a mean follow-up of 42.7 months.

## Methods

### Study subjects

This retrospective cohort study was conducted between May 2013 and March 2019. The children were referred for this study when refractory retinoblastoma was unresponsive with uncontrolled intravitreal seeding or tumour recurrence after receiving six cycles of systemic chemotherapy with a standard vincristine/etoposide/carboplatin (VEC) regimen in all cases and different strategies of local chemotherapy (IAC and/or IViC, Table 1). The children consecutively included were diagnosed with group D retinoblastoma, classified according to International Classification of Retinoblastoma (ICRB) criteria, with the other affected eye previously enucleated due to progressive retinoblastoma. All the included patients had no tumour involvement of the anterior segment or a suggestion of extraocular metastasis on neuroimaging. All of the cases are classified as cT2bN0M0H1 according to the 8th edition American Joint Committee on Cancer/Union for International Cancer Control Clinical Staging System (8th ed. cTNMH).

**Table 1:** Characteristics of patients with ICRB group D retinoblastoma underwent intravitreal chemotherapy-assisted endoresection

No.	Age (months)	Vitreous seeds Classification^a^	Subretinal seeds	Retinal detachment	TMN Classification^b^	Treatment history
01	36	Dust	Y	Y	cT2bN0M0H1	IVC + IViC
02	48	Dust	Y	Y	cT2bN0M0H1	IVC + IViC
03	8	Dust	Y	N	cT2bN0M0H1	IVC + IViC
04	30	Dust	Y	N	cT2bN0M0H1	IVC + IViC
05	24	Dust	Y	N	cT2bN0M0H1	IVC + IViC
06	78	Dust	Y	N	cT2bN0M0H1	IVC + IViC
07	71	Dust	Y	N	cT2bN0M0H1	IVC + IViC
08	65	Cloud	Y	N	cT2bN0M0H1	IVC + IViC
09	88	Cloud	Y	Y	cT2bN0M0H1	IVC + IViC
10	31	Spheres	Y	N	cT2bN0M0H1	IVC + IAC + IViC
11	29	Spheres	Y	Y	cT2bN0M0H1	IVC + IViC

*IVC* Intravenous chemotherapy, *IViC* Intravitreal chemotherapy, *IAC* Intra-arterial chemotherapy

^a^Vitreous seeding was classified by the criteria of Francis, J. H. et al. [11]

^b^According to the 8th edition American Joint Committee on Cancer/Union for International Cancer Control Clinical Staging System (8th ed. cTNMH)

### Treatment protocol

**Fig. 1:**
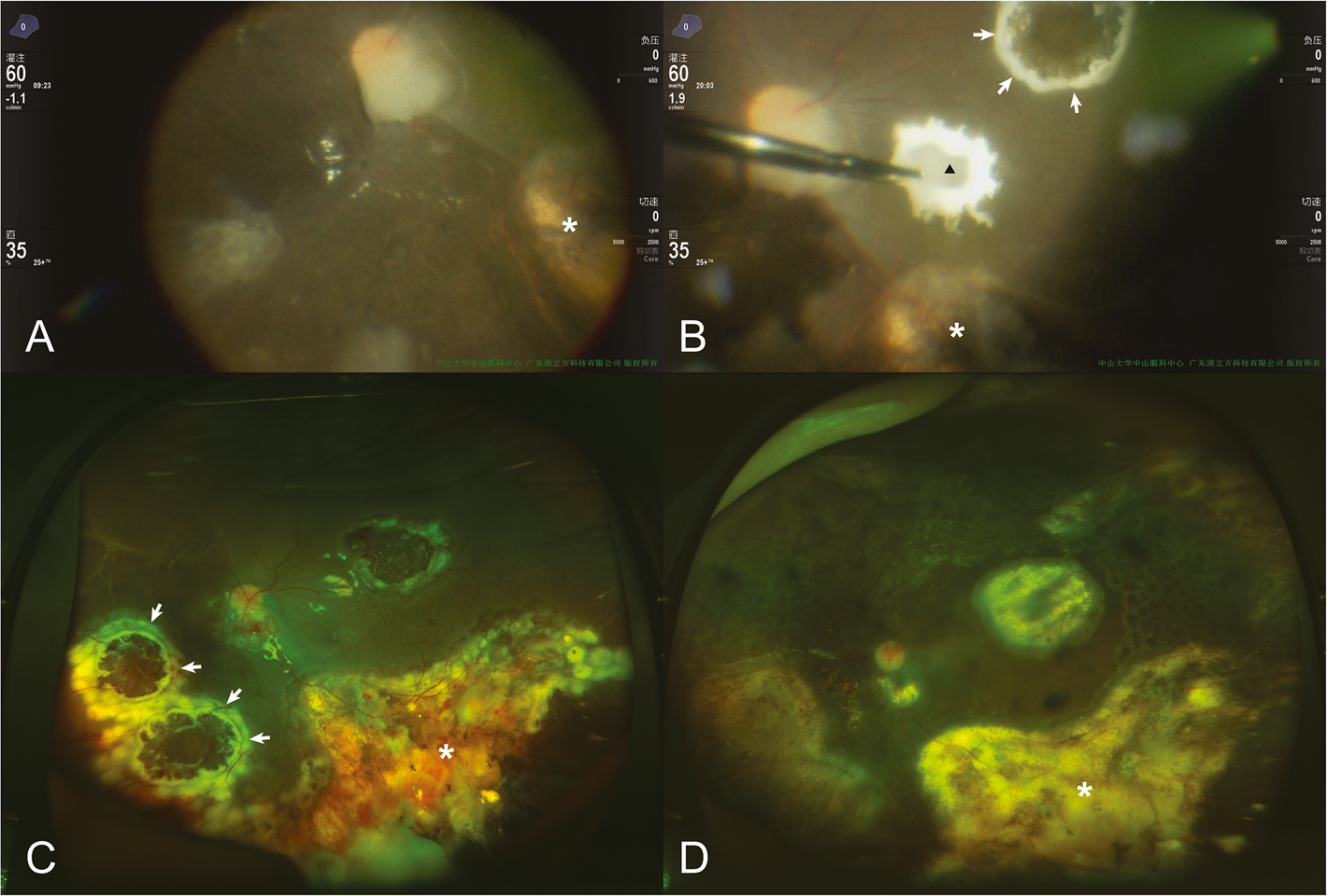
Chemotherapy-assisted endoresection of subretinal tumour in group D retinoblastoma in case 7. **a** and **b** The subretinal tumour was removed with extended resection by electrocoagulation (white arrows show the edge of electrocoagulation, black triangle shows the removed tumour and white asterisks show the scar of the regressed tumour after previous IViC). **c** One day after the endoresection, white arrows show the laser scarring around the resected lesion. **d** Sixteen months after endoresection and 1 month after silicone oil removal, no tumour recurrence was found (white asterisks show the scar of the regressed tumour)

**Fig. 2:**
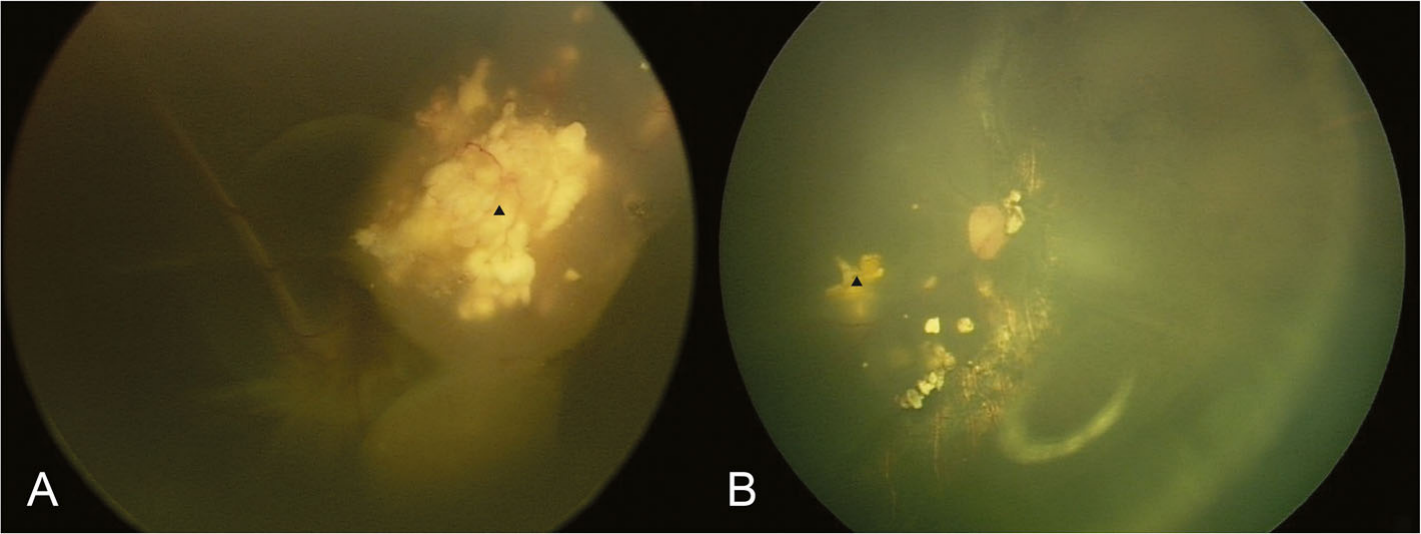
Reservation of calcified lesions during chemotherapy-assisted endoresection in group D retinoblastoma in case 11. **a** Refractory vitreous and subretinal seeds with massive calcification lesion in the only eye of a patient with bilateral retinoblastoma after IVC and IViC. **b** The resection of the tumour and the reservation of some calcified lesions, 1 month after surgery

IViC using topotecan (20μg/0.1 ml) was performed once, 1 week before surgery. While performing 25-gauge PPV, intraoperative IViC of 5 μg/ml topotecan in irrigation fluid (balanced salt solution) was applied. The visible intravitreal tumour cells were removed through vitrectomy; the subretinal tumour lesions were removed with extended resection accompanied by electrocoagulation at least 3 mm from the margin of the mass. To avoid retinal detachment, laser coagulation was applied to the boundaries after resection (Fig. 1). Cryotherapy was used when required. Calcified lesions were only removed if they were directly involved with the active tumour, or were located subretinally (Fig. 2). Silicone oil tamponade was applied in all cases. Intravitreal injection of topotecan (20 μg/0.1 ml) was applied at the end of the PPV. In all the cases, the lens was preserved during PPV.

Safety measures were undertaken to prevent orbital/extraocular extension of the tumour from the surgical incisions. All three incisions were sutured with absorbable 8–0 sutures to remain strictly watertight. Cryotherapy and sub-conjunctival injections of topotecan (20 μg/0.1 ml) were applied to all three of the sutured incision sites.

After surgery, all patients underwent routine eye examinations as well as neuroimaging (CT and MRI) to evaluate if there was any tumour recurrence, metastasis or orbital/extraocular extension. IViC and laser treatment were applied when there was any sign of recurrence. Additional PPV was performed when recurrence could not be controlled by IViC and/or laser treatment. Cataract surgery was performed for secondary cataract at least 6 months after the first PPV if there was no sign of recurrence or involvement of the anterior segment. Silicon oil removal was considered when any oil-related complications occurred in the eye, such as oil emulsification, oil migration, secondary glaucoma, etc.

This study was performed after obtaining informed consent from the patients and their family, and it was approved by the Board of Ethics at Sun Yat-Sen University Zhongshan Ophthalmological Center.

## Results

A total of 11 eyes were included in this retrospective cohort study conducted between May 2013 and February 2019. The basic information, ocular features and treatment history of the patients are presented in Table 1. The age of the patients ranged from 8 to 88 months, with an average age of 3.9 years. All of the patients were monocular, in which the other affected eye had been previously enucleated due to progressive retinoblastoma; the eyes included for combined therapy were classified as group D according to ICRB criteria. Subretinal seeding and vitreous seeding were observed in all the included cases. Vitreous seeding was classified according to the criteria by Francis et al [11]. No anterior segment or extraocular metastasis was found in any of the patients. Retinal detachment was observed in 4 patients (cases 1, 2, 9 and 11). Before referral to our hospital, all of the patients had previously received IVC and IViC; 1 patient (case 10) had also received IAC.

The follow-up time, tumour recurrence and metastasis, post-operative complications and subsequent treatments are presented in Table 2. Until March 2020, the follow-up time from PPV ranged from 12 months to 83 months, with a mean of 42.7 months. There was no case of anterior segment or extraocular tumour extension during follow-up period. Eye preservation was achieved in all 11 cases (100%). Vitreous and subretinal seeding control required PPV, once, in 9 cases (9/11, 81.8%), and no tumour recurrence was observed during follow-up.

**Table 2:** Treatment outcomes of patients who underwent chemotherapy-assisted endoresection

No.	Follow-up (months)	Recurrence	Metastasis	Complicated cataract	Retreatment	Pre-op BCVA	Last BCVA
01	83	No	No	No	No	HM	FC
02	81	No	No	No	No	HM	20/400
03	79	No	No	No	No	LP	20/200
04	65	No	No	No	No	LP	20/800
05	33	No	No	No	No	LP	20/100
06	30	No	No	No	Cataract surgery	LP	20/125
07	23	No	No	Yes	No	HM	20/400
08	23	Yes	No	Yes	PPV, IViC, cataract surgery	LP	20/200
09	23	Yes	No	Yes	Laser, IViC, PPV, Cataract surgery	HM	FC
10	18	No	No	Yes	Cataract surgery	LP	HM
11	12	No	No	No	No	LP	LP

*PPV* Pars plana vitrectomy, *IViC* Intravitreal chemotherapy

**Fig. 3:**
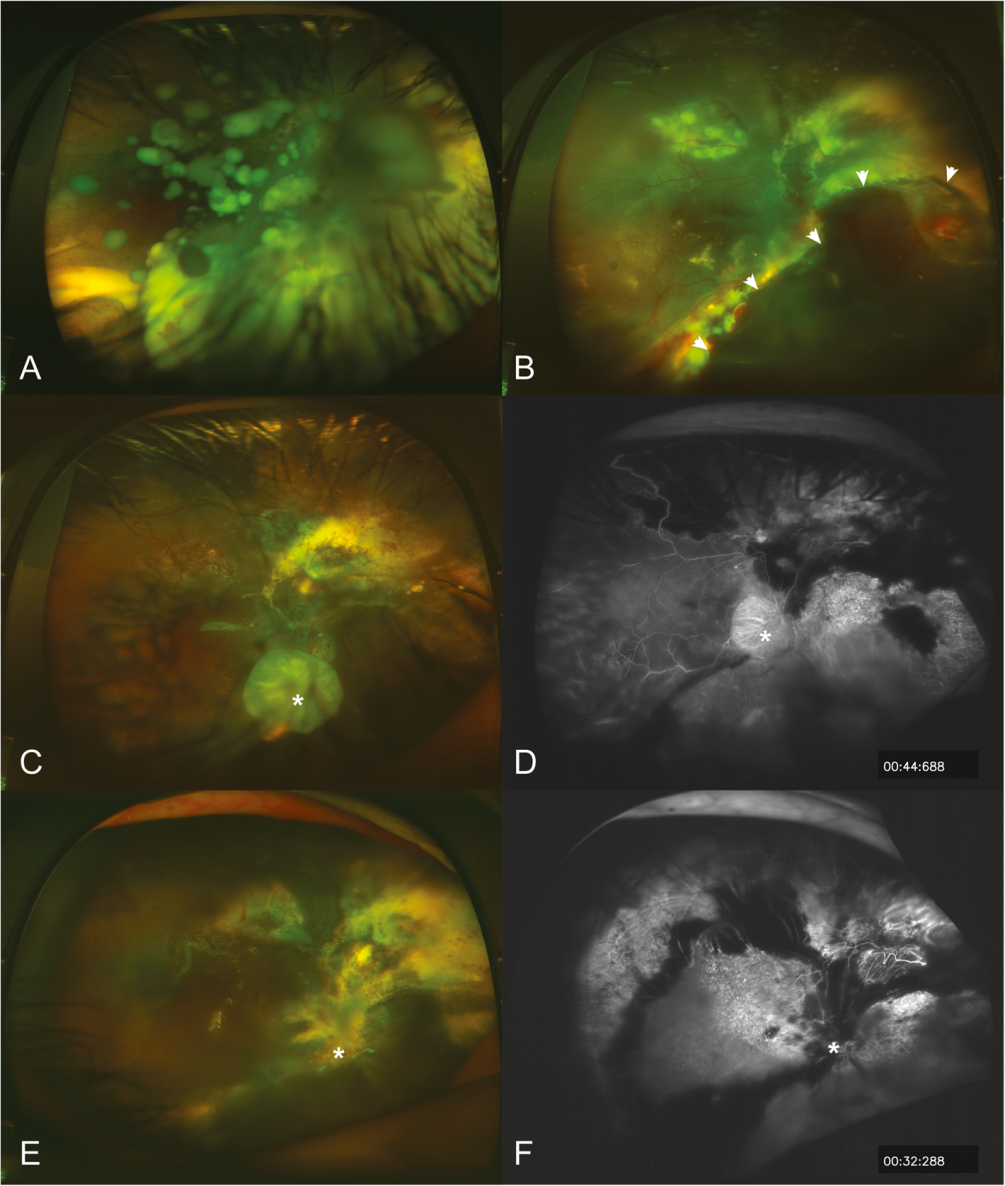
The management and result of focal tumour recurrence and retinal redetachment after chemotherapy-assisted endoresection in case 9. **a** Refractory vitreous and subretinal seeds with retinal detachment before chemotherapy-assisted endoresection. **b** White arrows show the edge of the resection of the tumour, 1 week after endoresection. **c** and **d** Focal tumour recurrence (white asterisk) with silicon oil tamponade. **e** and **f** Regression of the tumour (white asterisk) after 5 sessions of IViC and laser coagulation

Two cases (8 and 9) showed tumour recurrence after primary PPV without intravitreal seeding or anterior chamber involvement. In case 9, tumour recurrence was found 2 months after PPV, and it was observed as a focal solid tumour at the edge of previous endoresection. After repeated IViC (topotecan 20 μg/0.1 ml, every 2 weeks for 5 times) and retinal laser photocoagulation, the tumour regressed and no recurrence was observed during the follow-up period (Fig. 3). In case 8, tumour recurrence happened 12 months after PPV; it presented as multi-focal subretinal seeding with pre-retinal haemorrhage, without any vitreous seeding. After repeated IViC (topotecan 20 μg/0.1 ml, every 2 weeks for 3 times) and retinal laser photocoagulation, tumour growth could not be effectively controlled, so PPV with endoresection was performed again and no recurrence was observed by the end of the last follow-up.

Three of the 4 cases with pre-surgical retinal detachment achieved anatomical restoration of the retina after undergoing PPV once. One case (case 9) had recurrent retinal detachment 12 months after the first surgery, and retinal reattachment was achieved after a second PPV with no recurrent retinal detachment during subsequent follow-up. Secondary cataract was observed in 4 cases (cases 6, 8, 9 and 10) and cataract surgery (phacoemulsification and intraocular lens implantation) was performed. Silicone oil removal was performed in 2 cases (cases 6, 7) when silicone oil emulsification was seen. Silicon oil was not removed when focal recurrence was found (case 9); in this situation, further IViC and laser treatment were done in the silicon oil-filled eye.

The changes in best corrected visual acuity (BCVA) are shown in Table 2. Up to the last follow-up examination, 10 of the11 patients showed improvement in BCVA; in 4 cases the patient’s vision improved to ≥20/200. One patient (case 11) had no change in BCVA after PPV.

## Discussion

For many years, the risk of dissemination, seeding and extraocular spread has been the biggest obstacle for surgical intervention in patients with retinoblastoma. Honavar et al. reported an unfavourable outcome in 75% of patients with retinoblastoma who underwent PPV in 2001 [12]. With the rapid development of local chemotherapy treatments in recent years, this situation may come to an end. Several studies have reported on the combined use of IViC and PPV for advanced retinoblastoma in small sample cases [9, 10, 13]. Recently, Zhao et al. reported on planned PPV with IViC in 21 cases of refractory retinoblastoma, with eye preservation achieved in 85.7% (18/21) of the cases with a median of 5.1 years of follow-up [9]. In our study, eye preservation was achieved in 100% of the cases during an average follow-up of 42.7 months, without any seeding through the surgical tracts or metastasis. These encouraging results revealed that the combined use of IViC (both preoperatively and intraoperatively) significantly improved the safety of a surgical intervention for retinoblastoma. Moreover, to reduce the risk of surgical incision seeding, some safety measures should be taken, such as using a minimally invasive incision and applying strictly water-tight sutures, cryotherapy and subconjunctival injections of anti-tumour drugs at the incision sites. In general, we believe that IViC-assisted endoresection by PPV can be carefully considered as a supplementary therapy in the refractory cases when standard therapies have failed prior to second eye enucleation.

IViC-assisted endoresection should be performed with strict control of the indications and contraindications. We suggest that only previously treated (IVC/IAC/IViC) refractory ICRB group D cases with obvious vitreous and/or subretinal seeding should be considered for this form of treatment. The direct and definite removal of tumour is the significant advantage of endoresection, so it is especially helpful for cases with a high burden of vitreous and/or subretinal seeding. The IViC-assisted endoresection by PPV can reduce the tumour size and its resulting burden while facilitating extensive and uniformed distribution of the chemotherapeutic drug in the vitreous cavity, which further enhances the efficiency of the drug and reduces the amount of treatment required and the retinal toxicity of repeated IViC [6].

The contraindication of endoresection for retinoblastoma is any sign of tumour metastasis in the anterior segment or extraocular metastasis, which suggests that ICRB group E cases should be excluded. In the study by Zhao et al., the retinoblastoma was not controlled by one-time use of PPV in 4 patients, all of whom demonstrated tumour metastases in the anterior chamber of the eyes, and only one eye was preserved [9]. This result is consistent with the findings reported in many previous studies that anterior chamber tumour metastasis is a danger sign that implies poor prognosis and a high risk of recurrence and extraocular metastasis [14, 15]. Moreover, in these eyes, PPV may greatly increase the risk of iatrogenic seeding because the incision is very close to the anterior segment of the eye. Consequently, we also suggest lens-preserving PPV even when there is partial opacity of the lens, in order to minimise disturbance to the anterior segment during surgery and lower the risk of iatrogenic seeding to the anterior segment. It should be noted that secondary cataract might develop due to silicone tamponade or chemotherapeutic drug toxicity after PPV. In our opinion, cataract surgery should be cautiously considered at least 6 months after PPV when there is no sign of tumour recurrence or metastasis.

Although many preventive measures have been used, the tumour can still recur. In our study, two cases had single or multiple focal subretinal tumour recurrence. Fortunately, the recurrent tumours could be eradicated by repeated IViC, laser coagulation or a second PPV. The relatively good prognosis was mostly attributed to the absence of recurrent vitreous seeding. We assumed the silicone oil tamponade helped confine the tumour as a focal lesion of the retina, thus limiting intravitreal tumour seeding. Therefore, intravitreal silicone oil tamponade is recommended during PPV to prevent tumour dissemination, especially vitreous seeding. Since the tumour may recur a long time after surgery (e.g. in case 8, tumour recurrence was observed 12 months after PPV), we suggest that the silicone oil tamponade time should be prolonged appropriately, unless any oil-related complications occur.

In addition to eradicating intraocular tumours, IViC-assisted PPV can also help address the sight-threatening vitreoretinal complications of retinoblastoma, such as vitreous haemorrhage and persistent retinal detachment. In our study, 4 of the11 eyes had pre-surgical retinal detachment, and all of them achieved successful anatomical retinal reattachment after PPV. This is especially crucial for monocular patients who have no other alternative for retaining visual function and who require eye preservation. In our study,10 of the 11 monocular patient had improved vision after PPV; in 4 cases, the patient’s vision had improved to ≥20/200. The favourable visual function outcomes also proved the value and good prospect of this treatment strategy.

However, this study has some limitations. Like any new technique, it is difficult to conduct a randomised controlled trial for treating retinoblastoma, as it is a relatively rare disease. The follow-up period in this study was also relatively short, with a mean of 42.7 months (ranging from 12 months to 83 months). Since retinoblastoma could recur years later, further follow-up is required to evaluate the long-term effects of this treatment method. We also used topotecan instead of melphalan because topotecan was the only chemotherapy drug currently approved in mainland China for the treatment of retinoblastoma.

## Conclusions

This study showed encouraging results for using IViC-assisted endoresection to control ICRB group D retinoblastoma. This suggests that it might be considered to be a supplementary therapeutic strategy for some refractory cases that are unresponsive to standard therapies, especially for monocular patients for whom the other affected eye has been previously enucleated.

## Data Availability

The datasets used during and/or analysed in this study are available from the corresponding author upon request.

## Abbreviations

ICRB: International Classification of Retinoblastoma
IVC: Intravenous chemotherapy
IAC: Intra-arterial chemotherapy
IViC: Intravitreal chemotherapy
PPV: Pars plana vitrectomy
VEC: Vincristine/etoposide/carboplatin.
BCVA: Best corrected visual acuity
